# Lung Cancer Onset in Wild Type Mice Following Bone Marrow Reconstitution with *kras*^v12^ Cells

**DOI:** 10.1038/srep13047

**Published:** 2015-08-12

**Authors:** Elena Belloni, Ines Martin Padura, Elvira Gerbino, Stefania Orecchioni, Fulvia Fusar Imperatore, Paola Marighetti, Giovanni Bertalot, Pier Giuseppe Pelicci, Francesco Bertolini

**Affiliations:** 1 European Institute of Oncology, Department of Experimental Oncology, Milan, Italy; 2 European Institute of Oncology, Laboratory of Hematology-Oncology, Milan, Italy; 3 European Institute of Oncology, Molecular Medicine for Care Program, Milan Italy; 4 Dipartimento di Scienze della salute, Università degli Studi di Milano, Milan, Italy

## Abstract

A role for bone-marrow-derived cells (BMDCs) in tissue repair and malignancy onset has been proposed, but their contribution is still debated. We tested the ability of BMDCs containing the inducible *kras*^V12^ oncogene to initiate lung adenocarcinoma. For our experimental strategy, we reconstituted lethally irradiated wild type mice with BMDCs carrying inducible *kras*^V12^ and subsequently induced oncogene expression by 4-OHT administration. Epithelial lung lesions, from adenoma to adenocarcinomas, appeared at successive time points. These results show that lung tumors were derived from donor BMDCs and indicate a direct involvement of bone marrow cells in the development of epithelial cancers.

Oncogenic *KRAS* mutations are detected in 10 to 30% of non-small-cell lung cancers (NSCLCs, mostly adenocarcinomas in heavy-smokers patients)^1^, and are usually associated with a poor prognosis^2^.

A mouse model expressing oncogenic krasV12 (*kras*^+/V12^*RERT*^ ert/ert^, generated in the laboratory of Mariano Barbacid^3^,^4^) recapitulates the neoplastic phenotype induced by the *kras*^V12^ mutation. Severe lung lesions appear upon oncogene induction by 4-hydroxitamoxifen (4-OHT), evolving from hyperplasia, through adenoma, to adenocarcinoma. Mice develop lung tumors with complete penetrance. Additional lesions (histiocytic sarcomas, sarcomas and anal papillomas) are detected in only 30% of the animals and no other tumor type is observed.

Studies have indicated a possible role for bone-marrow-derived cells (BMDCs) in lung injury repair, and a population of BMDCs expressing the lung-specific Ccsp marker has been isolated. After injection, these cells are capable of homing to the lung and differentiating into multiple epithelial cell lineages. Their retention in the lung is more efficient after naphthalene injury, highlighting a possible role in lung tissue repair and regeneration^5^,^6^.

BMDCs are frequently recruited to sites of tissue injury and inflammation and can differentiate into parenchymal cells of various non-hematopoietic tissues, including lung. However, it is unclear what contribution they make to epithelial regeneration^7^. Adult BMDCs are able, albeit rarely, to engraft into injured lungs of *Cftr* (cystic fibrosis transmembrane conductance regulator) knock-out mice and differentiate into functional airway epithelial cells, displaying *Cftr* mRNA and protein expression^8^.

A potential role for BMDCs in malignancy has also been hypothesized^9^, and a recent study^7^ investigated their contribution during the onset of induced squamous cell carcinoma (SCC). Another study explored the possible role of BMDCs in the pulmonary carcinogenic process using a double mutant *kras*^G12D^*p53*^fl/fl^ mouse model^10^.

To explore the mechanisms underlying the interactions between BMDCs and tumor cells, we tested the ability of bone marrow cells containing an inducible oncogene to initiate lung adenocarcinoma. After irradiation, wild-type (wt) mice transplanted with donor bone marrow cells derived from the *kras*^+/V12^*RERT*^ ert/ert^ (*kras*^V12^) mouse model developed lung tumors upon induction of the oncogene.

## Results

Bone marrow cells were obtained from *kras*^V12^ mice and transplanted into 18 irradiated wt recipient mice (mice are summarized in Supplementary table 1; five died soon after 4-OHT treatment and were not considered). Eight of the transplanted mice were subsequently treated with 4-OHT to induce the *kras*^V12^ oncogene, while the remaining five untreated mice were used as controls. Mice were monitored and sacrificed at subsequent post-induction time points (0, 3.5, 5.5, 8.5, 11, 13.5, 15, and 16 months). Lungs, spleen, liver, femur and peripheral blood were collected. Blood parameters (summarized in Supplementary table 2) as well as blood smears (data not shown) were analyzed at different time points, when possible, in order to verify bone marrow reconstitution and check whether the oncogene induction could result in variations on blood cells counts. No relevant abnormalities were detected.

**Figure 1:**
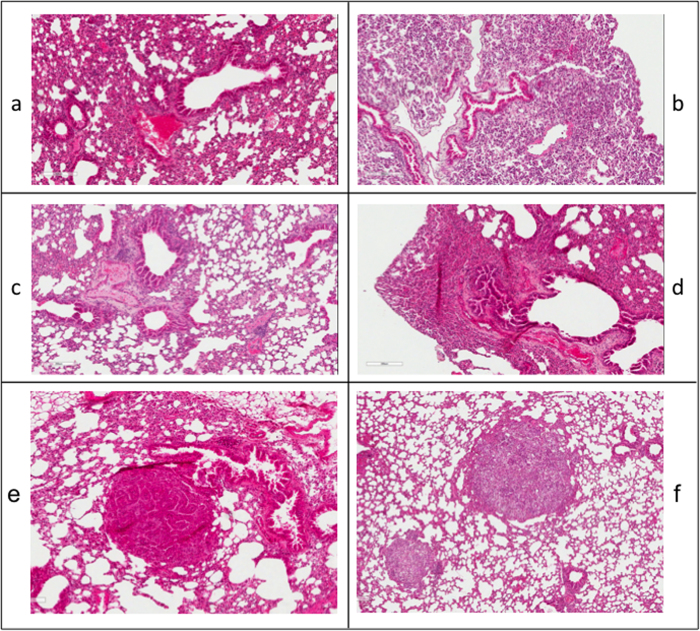
Effect of *kras*^V12^ bone marrow cells reconstitution on the lung of 4-OHT untreated and treated mice. **a-d**): H&E staining of lung tissue sections from *kras*^V12^-reconstituted mice without oncogene induction. Images represent four consecutive time points: a = 0, b = 3.5, c = 8.5, d = 11 months**. e-f**) Lesions in lung sections from reconstituted mice after 4-OHT oncogene induction. H&E staining: **e**) papillary adenomas (3.5 months post-induction) and **f**) solid adenocarcinoma foci (5.5 months post-induction).

Bone marrow reconstitution was checked by PCR for the *βGEO* gene on DNA extracted from peripheral blood of the transplanted mice (Supplementary figure 1). Analysis of the H&E-stained lung sections from reconstituted *kras*^V12^ and wt mice revealed diffuse hyperplasia, possibly due to irradiation (Fig. 1a–d). Epithelial lesions, adenomas and/or adenocarcinomas, were detected in 4 out of 8 *kras*^V12^-reconstituted animals at 3.5, 5.5, 13.5 and 15 months post oncogene induction (Fig. 1e–f). No lesions were detected in non-induced *kras*^V12^-reconstituted animals. βgal staining of lung lesions in reconstituted mice confirmed expression of the *βGEO* marker, and therefore of the *kras*^V12^ oncogene (Supplementary figure 2). As expected, irradiated *kras*^V12^ mice reconstituted with wt BMDCs also developed lung lesions upon 4-OHT oncogene induction (data not shown).

Lesions were analyzed by immunohistochemistry (IHC) with an antibody specific for pan-cytokeratin (pan-ck, a marker for epithelial cells) and by immunofluorescence (IF) with antibodies specific for type II pneumocytes (spc) and club cells (cc10), the major epithelial cells types of alveoli and bronchi/bronchioles, respectively. As seen for the *kras*^V12^ mouse model, lung lesions detected in the *kras*^V12^ bone marrow reconstituted animals express pan-ck and only the alveolar spc protein (Supplementary figure 3).

## Discussion

In the present study, we show that lung tumors develop in wt animals, reconstituted with BMDCs harboring the *kras*^V12^ oncogene.

BMDCs are known to be recruited to sites of tissue injury or inflammation, however, the potential of recruited BMDCs carrying an oncogene to act as a source of malignancy was disputed for a long time. This potential was first reported for the onset of gastric cancer of donor origin^9^, and other cases have since been presented^11^, confirming that marrow donor cells contribute to solid tumors.

Further work has shown that the bone marrow cell population recruited to parenchymal tissues includes mesenchymal stem cells (MSCs)^10^, which become resident stromal cells. MSCs are implicated in the formation of tumor-associated stroma and facilitate tumor progression^12^. Our current findings suggest that MSCs recruited to the injured organ not only have the potential to form part of the tumor associated stroma, but might also be the initiating tumor cells upon activation of the oncogene. We are currently investigating whether the tumor-initiating potential of BMDCs is higher in MSCs or in hematopoietic cells. Indeed, a case of endobronchial inflammatory myofibroblastic tumor (IMT), a low-grade mesenchymal tumor, was recently reported in a child following allogeneic bone marrow transplantation^13^.

Upon krasV12 activation, 4 out of 8 induced mice did not develop lung lesions. Due to the small number of animals analyzed in the present study, we could only speculate that lesions in these mice would have appeared later in time or, alternatively, that an incomplete bone marrow reconstitution was obtained in some of the transplanted mice, with a consequent partial or delayed development of the corresponding phenotypes. Finally, it could also be possible that a second hit would be necessary for the onset of lung neoplasia following oncogene induction in krasV12 bone marrow reconstituted animals, and that such an event did not occur in all the treated animals.

Of note, evaluation of blood parameters is unfortunately not fully able to show whether the oncogene induction could result in a phenotype on hematopoietic cells. In fact, it is a quantitative representation of immune cells, which could be affected by different factors. To solve this matter in future experiments, we will investigate cancer promoting cytokines/chemokines, and specific immune cell markers.

Our results emphasize the direct implication of BMDCs in lung epithelial tumor development. Further studies are warranted to dissect the mechanisms that regulate BMDCs’ recruitment to injured tissues and their role in tumor initiation and/or progression.

## Methods

### Ethics statement

Experiments involving animals have been done in accordance with the Italian Laws (D.L.vo 116/92 and following additions), which enforced the EU 86/609 Directive (Council Directive 86/609/EEC of 24 November 1986 on the approximation of laws, regulations and administrative provisions of the Member States regarding the protection of animals used for experimental and other scientific purposes). Mice have been housed accordingly to the guidelines set out in Commision Recommendation 2007/526/EC - June 18, 2007 on guidelines for the accommodation and care of animals used for experimental and other scientific purposes.

Until March 28, 2014, the Italian legislation did not require a specific ethical review process for all the experiments involving animals. A central (Government) review was required only for particular species (e.g., dogs, cats, and non-human primates) or for experiments done without anesthesia or that will or may cause severe pain. In the other cases, only a notification of the experiments to the Ministry of Health was required. Accordingly, the project was notified to the Ministry of Health.

### Oncogene induction in *kras* ^*V12*^ mice

The *kras*^*V12*^ mouse model that we have imported in our laboratory is based on a bi-cistronic transgene, encoding the *kras*^*V12*^oncogene and the *β geo* marker. Both *kras*^V12^ and *β geo* can be induced by administration of 4-hydroxitamoxifen (4-OHT). 4-OHT (0.5 mg per dose, 3 doses per week, for 2 weeks) was injected intraperitoneally (ip). Time-course lung tissue examination - following hematoxylin/eosin (H&E) staining - revealed lung lesions of increasing severity: from hyperplasia (at 2–3 months post-induction), to adenoma (4–5 months post-induction), to adenocarcinoma (6–9 months post-induction).

### Bone marrow cells transplantation

Bone marrow cells were isolated from six donor *kras*^V12^ mice or wt controls. For bone marrow transplantation, donor mice were euthanized by CO_2_ asphyxiation according to institutional guidelines. Bone marrow cells were harvested by crushing the femurs and tibias with a mortar and pestle in RPMI 1640 medium (Euro Clone) containing 50 μg/ml gentamicin (Gibco). Bone marrow cells were filtered through a 40-μm-nylon cell strainer. The cell suspension was then pelleted at 1,500 rpm for 5 minutes, resuspended, and counted. A total of 10 × 10^6^ wt bone marrow cells was transplanted intravenously (iv), through the lateral tail vein into 6-to-8-weeks old wt recipient mice (18 recipients in total) that had been lethally irradiated with 8 Gy 24 hours earlier. Repopulation by the transplanted bone marrow cells was confirmed by PCR of the *βGEO* gene from DNA extracted from peripheral blood using the Illustra Blood GenomicPrep Mini Spin Kit (GE Healthcare). A 108 bp PCR product was obtained with a DNA to a PCR cycle of denaturation at 95 ^o^C for 5 min, followed by 30 cycles of denaturation at 95 ^o^C for 1 min, annealing at 60 ^o^C for 30 sec and extension at 68 ^o^C for 1 min, and a final single elongation cycle at 72 ^o^C for 5 min. The following *βGEO*-specific primers were used: forward (5′-TACGCCAATGTCGTTATCCA-3′); reverse (5′-CGTCAGTCAGGCCTTCTTTC-3′). Mice were monitored and sacrificed at successive time points (0, 3.5, 5.5, 8.5, 11, 13.5, 15, and 16 months). Lungs were removed and either snap-frozen in OCT or formalin-fixed and paraffin embedded. When possible, other tissues (femurs, spleen, liver) were collected, together with blood samples for blood smears and blood cell counts.

### Hematoxylin and eosin (H&E) staining

Lungs were harvested from mice at successive time points, formalin fixed, and paraffin embedded. Lung tissue sections (5μm) were dewaxed, rehydrated, and then stained with H&E. After staining and dehydration, glass sections were mounted in Eukitt mounting medium (Sigma) and then scanned with a Scanscope XT system (Aperio technologies, Inc). Images were acquired with ImageScope software (Aperio technologies, Inc).

### *βgal* staining

Lungs were harvested from mice at different time points and included in OCT. Frozen samples were cut into 5 μm slices and stained for beta-galactosidase as follows. Tissue sections were fixed with 0.2% glutaraldehyde, washed several times in buffer containing 0.1 M NaH_2_PO_4_, 2 mM MgCl_2_, 0.01% Na deoxycholate, and 0.02% Nonidet, and incubated in a solution of 5 mM K_3_Fe(CN)_6_, 5 mM K_3_Fe(CN)_6_H_2_O and 1 mg/ml X-Gal at 37 ^o^C for 24–48 h. Slides were then washed and counterstained with nuclear fast red, mounted in Eukitt mounting medium (Sigma), and then scanned with a Scanscope XT system (Aperio technologies, Inc). Images were acquired with ImageScope software (Aperio technologies, Inc).

### Immunofluorescence

Immunofluorescence was performed with spc- and cc10-specific antibodies, according to the following procedure. Paraffin sections (5 um) of lung tissue were pretreated for antigen-retrieval with Sodium Citrate buffer, pH 6, followed by cooling for 20 mins to room temperature. Subsequent to blocking with 2% bovine serum albumin in PBST, sections were incubated with antibodies against lung cell-type-specific marker proteins: rabbit Anti-Prosurfactant Protein C (pro SPC, 1:1000, AB3786 Millipore) and goat Anti-cc protein 10 (CC10, 1:400 Santa Cruz biotech., INC.). The antigen-antibody complexes were visualized by commercially available fluorescently labeled secondary antibodies: anti-rabbit IgG Alexa 647 (1:100, Invitrogen) and anti-goat IgG-Cy3 (1:400, LiStarFish). Sections were then incubated with DAPI (1:5000, D9564 Sigma-Aldrich), fixed with 2% PFA and mounted in mowiol mounting medium.

### Immunohystochemistry

Immunohistochemistry was performed with a pan-ck-specific antibody, according to the following procedure.

Paraffin sections (5 um) of lung tissue were pretreated for antigen-retrieval with Sodium Citrate buffer, pH 6, followed by cooling for 20 mins to room temperature. After blocking the endogenous peroxidase with 3% H_2_O_2_ on specific protein binding sites, lung sections were first blocked for 1 h with 2% BSA in TBST (TBS, 0.05% Tween20) and then incubated with the primary monoclonal mouse Anti-PanCytokeratin (1:400, C2562 Sigma Aldrich) overnight at 4 ^o^C. The detection of antigen-antibody complexes was obtained with a HRP conjugated anti-mouse secondary antibody (ready to use, K4001 DAKO) and subsequent visualization with a chromogen system (Liquid DAB + substrate, K3468 DAKO). The sections were finally counterstained with hematoxylin for 1′, dehydrated in a series of ethanol and xylene and mounted in Eukitt mounting medium.

## Additional Information

**How to cite this article**: Belloni, E. *et al*. Lung Cancer Onset in Wild Type Mice Following Bone Marrow Reconstitution with *kras*^V12^ Cells. *Sci. Rep*. **5**, 13047; doi: 10.1038/srep13047 (2015).

## Supplementary Material

Supplementary Information
