# Supplementary material for: Lung Cancer Onset in Wild Type Mice Following Bone Marrow Reconstitution with krasv12 Cells
Source: Sci Rep. 2015 Aug 12;5:13047. doi: 10.1038/srep13047 (PMC4533313; doi:10.1038/srep13047)
Supplement: Supplementary Information [file srep13047-s1.pdf]

Supplementary information

LUNG CANCER ONSET IN WILD TYPE MICE FOLLOWING BONE MARROW RECONSTITUTION WITH *k-ras*^{V12} CELLS.

Elena Belloni[1*], Ines Martin Padura[2#*], Elvira Gerbino[1], Stefania Orecchioni[2], Fulvia Fusar Imperatore[1], Paola Marighetti[2@], Giovanni Bertalot[3], Pier Giuseppe Pelicci[1,4], and Francesco Bertolini[2].

1. European Institute of Oncology, Department of Experimental Oncology, Milan, Italy.

2. European Institute of Oncology, Laboratory of Hematology-Oncology, Milan, Italy.

3. European Institute of Oncology, Molecular Medicine for Care Program, Milan Italy.

4. Dipartimento di Scienze della salute, Università degli Studi di Milano, Milan, Italy.

[#] Present Address: Spanish National Center for Cardiovascular Diseases (CNIC), Madrid, Spain

[@] Present Address: Snyder Institute for Chronic Diseases, University of Calgary, Department of Physiology and Pharmacology, Calgary, Canada

[*]These authors equally contributed to this work.

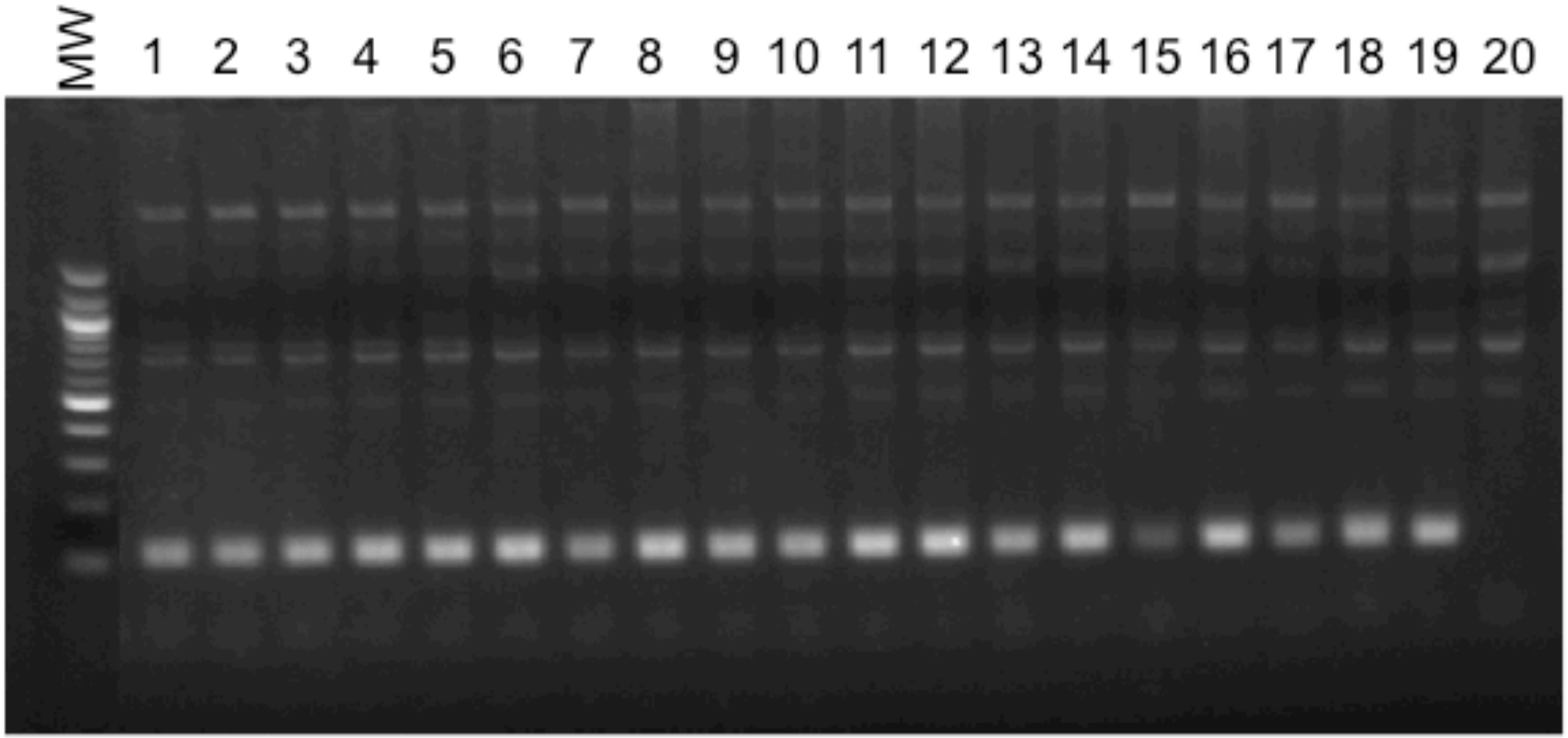

Supplementary figure 1

Legend to supplementary figure 1. PCR results for amplification of the *βgeo* gene in reconstituted mice.

Repopulation by the transplanted bone marrow cells was confirmed through PCR of the *βGEO* gene: results in the 18 reconstituted mice are shown. Lane IDs are as follows: MW=100bp molecular weight marker, 1=13960, 2=13961, 3=13962, 4=13963, 5=13968, 6=14513, 7=14514, 8=14736, 9=14737, 10=14738, 11=14740, 12=14741, 13=14743, 14=14748, 15=14752, 16=15124, 17=15125, 18=15126, 19=$kras^{V12}$ reference mouse, 20=wt reference mouse. Mice # 2=13961, 3=13962, 4=13963, 5=13968 and 10=14738 died soon after 4-OHT induction and were not further considered.

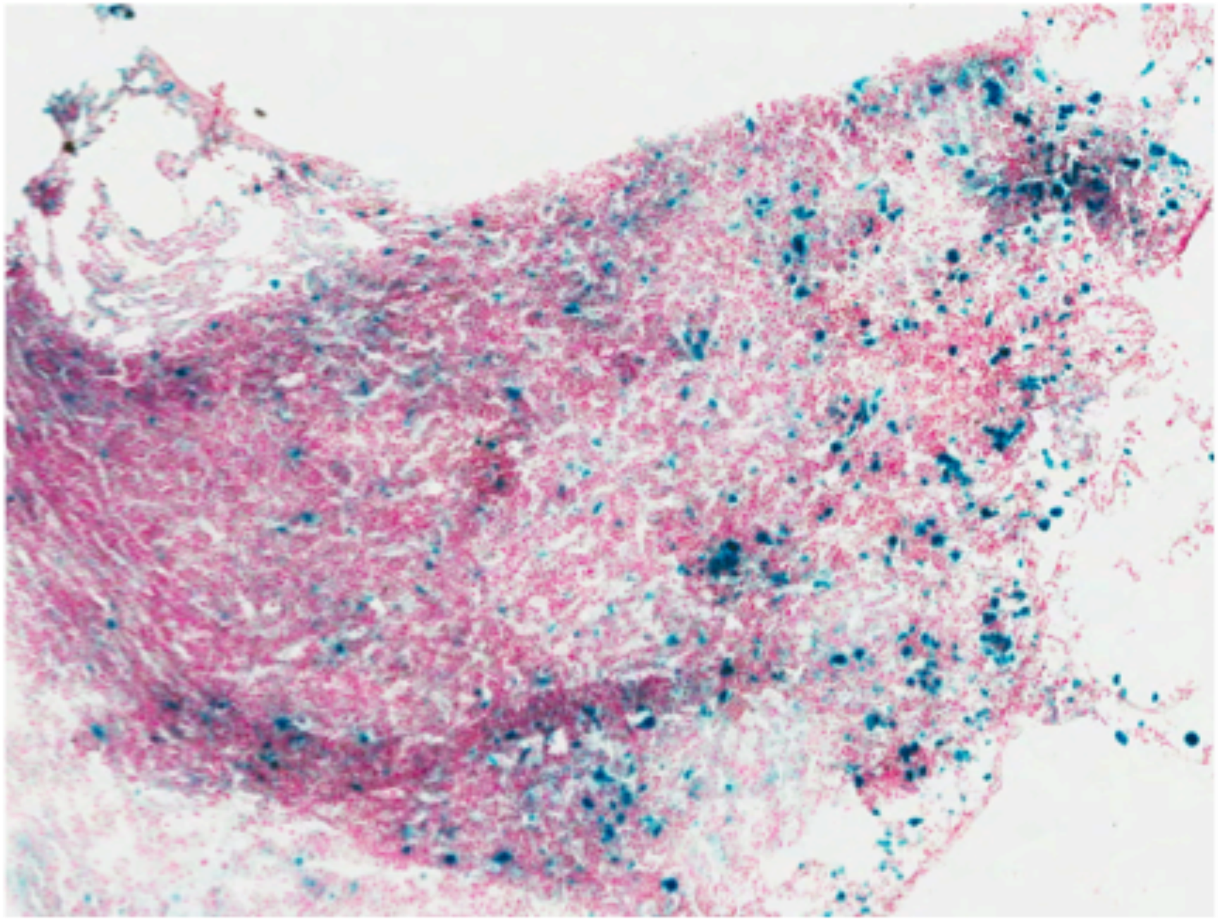

Supplementary figure 2

Legend to supplementary figure 2. Expression of the *βgeo* marker.

βgal staining of a lung tissue section from a mouse reconstituted with $kras^{V12}$ bone marrow cells after 4-OHT oncogene induction (time point: 15 months post 4-OHT induction).

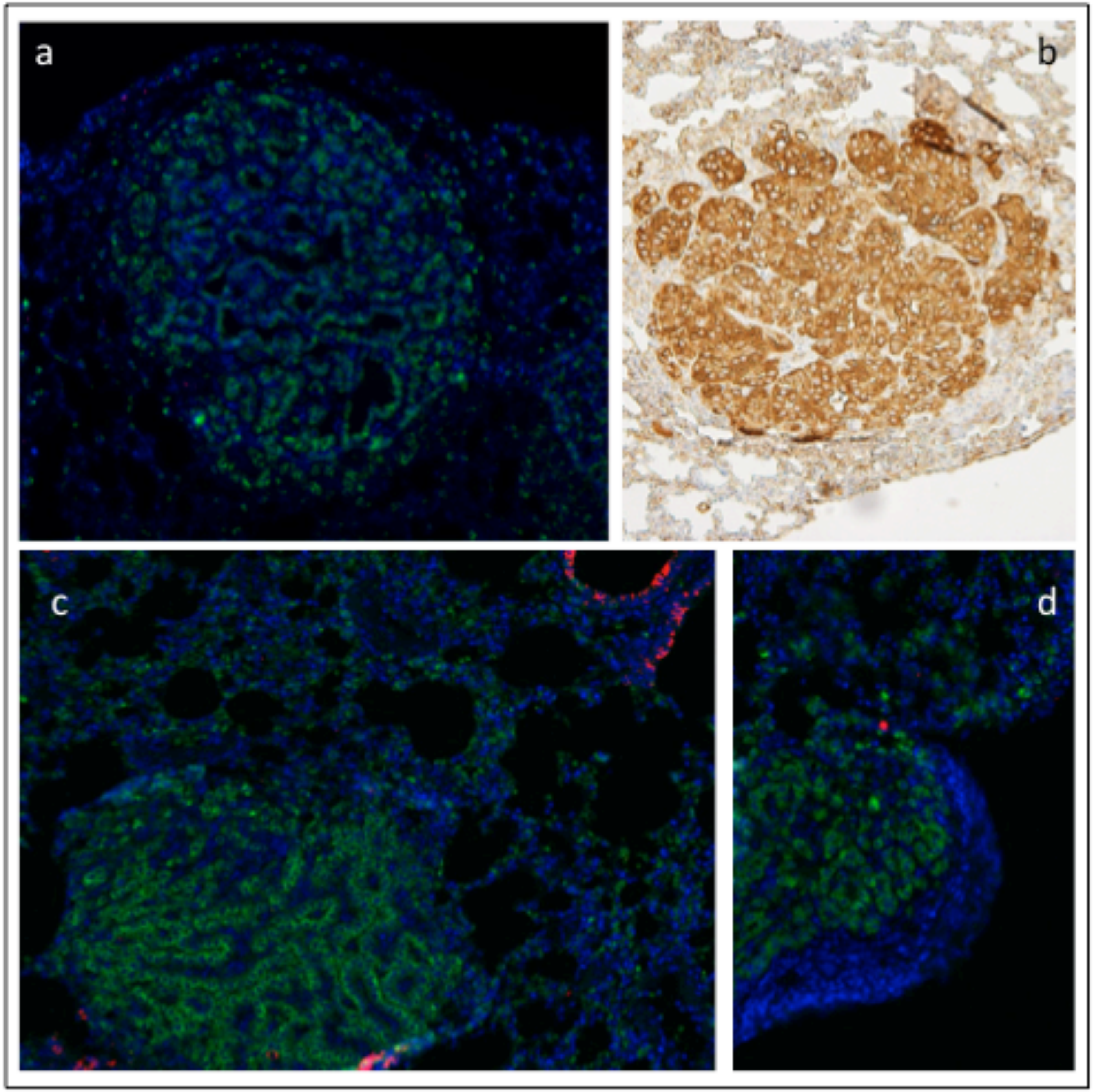

Supplementary figure 3

Legend to supplementary figure 3. Expression of epithelial markers.

Expression of the pan-ck (epithelial), spc (alveolar), and cc10 (bronchiolar) proteins was checked by either IHC or IF, with specific antibodies on lung sections from *kras*^{V12}, as well as *kras*^{V12} bone marrow reconstituted mice. a = IF for spc and cc10 on a lung lesion detected in a *kras*^{V12} mouse, 7 months post 4-OHT induction; b = IHC for pan-ck on a lung lesion detected in a *kras*^{V12} bone marrow reconstituted mouse, 5.5 months post 4-OHT induction; c-d = IF for spc and cc10 on a lung lesion detected in a *kras*^{V12} bone marrow reconstituted mouse, 3.5 and 13.5 months post 4-OHT induction, respectively). [Green = spc; red = cc10].

NB: in panel c, cc10-positive staining can be detected outside the adenoma lesion, lining a bronchiolus, as expected.

Supplementary table 1

**Mouse #** (induced [*control*])	**Months**	**Lesions**
[14752]	0	[NO]
15124 [*14513*]	3.5	YES [NO]
14740	5.5-1	YES
13960	5.5-2	NO
14737 [*14741*]	8.5	NO [NO]
15125 [*14514*]	11	NO [NO]
14736	13.5	YES
14743 [*15126*]	15	YES [NO]
14748	16	NO

Legend to Supplementary table 1. Summary of results from *krasV12* reconstituted mice.

18 wt irradiated mice were used as recipients: five died following 4-OHT induction and were not further considered. The remaining 13 are listed in the table. First column: mice IDs, controls are indicated *in italic* between square brackets; second column: post-induction time points; third column: presence of lung lesions (YES/NO).

Supplementary table 2

	Ref. min	Ref. max	14752<br>Ctrl 0m	14513<br>Ctrl 3.5m	15124<br>4-OHT 3.5m	14740<br>4-OHT 5.5m	14741<br>Ctrl 8.5m	14737<br>4-OHT 8.5m	15125<br>4-OHT 11m	14736<br>4-OHT 13.5m	14743<br>4-OHT 15m	14748<br>4-OHT 16m
**WBC** ($10^3/\mu l$)	1.8	10.7	1.1	6.5	5.5	7.3	11.3	5.3	6.8	8.4	3.3	0.8
**NEU** ($10^3/\mu l$)	6.6	38.9	21.6	6.6	6.5	7.3	2.6	1.8	2.4	4	2.6	18.6
**LIM** ($10^3/\mu l$)	55.8	91.6	62.7	69.1	92.1	90	95.9	96.2	92	95.2	95.6	72.3
**BAS** ($10^3/\mu l$)	0	2	6.8	9.1	0.5	1.1	0.4	0.3	0.8	0.3	0.3	3.1
**RBC** ($10^3/\mu l$)	6.36	9.42	4.73	1.11	8.95	4.51	5.55	4.08	3.36	6.1	4.77	1.98
**HGB** (g/dl)	11	15.1	6.4	1.8	11.3	7.1	7.3	5.8	6.1	7.2	7.9	3.9
**PLT** ($10^3/\mu l$)	592	2972	137	33	597	754	+++++	+++++	785	1195	619	304

Legend to Supplementary table 2. Summary of blood parameters.

Blood parameters were available for some of the mice included in the study (mice IDs are indicated). First two columns refer to minimum and maximum levels for each parameter in wt normal mice. Abbreviations are as follows: WBC = white blood cells; NEU = neutrophils; LIM = lymphocytes; BAS = basophils; RBC = red blood cells; HGB = hemoglobin; PLT = platelets. +++++ indicates out of scale values.
